# Soziale Spaltung und Corona-Kapitalismus: Kontexte für Kinderrechte und (Kinder‑)Armut

**DOI:** 10.1007/s12054-020-00343-x

**Published:** 2020-12-15

**Authors:** Michael Klundt

**Affiliations:** Hochschule Magdeburg-Stendal, Magdeburg, Deutschland

**Keywords:** Soziale Spaltung, Kinderrechte, Armut, Covid-19, Pauperisierung

## Abstract

Der Beitrag untersucht zunächst die sozioökonomischen Bedingungen der sozialen Spaltungsprozesse im Corona-Crash, um daraufhin Implikationen für Kinderechte und (Kinder-)Armut in einem reichen Land zu betrachten.

Wer über soziale Spaltung spricht, darf sich nicht nur mit Armut und sozialer Benachteiligung beschäftigen. Erst recht in sehr reichen Gesellschaften wie Deutschland muss auch über den riesigen gesellschaftlichen Wohlstand gesprochen werden, der zwar von allen erwirtschaftet, aber nur von ganz wenigen angeeignet und bestimmt wird. Aus dieser Analyse heraus lässt sich erkennen, wie diese Gesellschaft in den Corona-Crash hineingegangen ist und wie sie dort wieder herausausgeht.

Dass Corona und die Maßnahmen dagegen nicht auf alle Alters- und Sozialgruppen gleich wirk(t)en, scheint inzwischen eine Binsenweisheit (vgl. Bertelsmann Stiftung 2020). Gerade für Kinder und vor allem für Minderjährige in Armut bedeuteten Corona und der Lockdown enorme Einschränkungen ihrer zivilen, sozialen und kulturellen Rechte auf Schutz, Förderung und Beteiligung – besonders in den Bereichen Kindeswohl, Gesundheit, Bildung (vgl. Spieß 2020; United Nations 2020; UNICEF/Save the Children 2020; UN-Ausschuss für die Rechte der Kinder 2020; Kinderkommission des Deutschen Bundestages 2020). Entsprechend untersucht der Beitrag zunächst die sozioökonomischen Bedingungen der sozialen Spaltungsprozesse im Corona-Crash, um daraufhin Implikationen für Kinderechte und Kinder in Armut zu betrachten.

## Erscheinungsformen sozialer Spaltung in der Corona-Krise

Laut der globalisierungskritischen Nichtregierungs-Organisation Oxfam vom September 2020 seien „weltweit während der Pandemie etwa 400 Millionen Jobs verloren gegangen (…) und bis zu einer halben Milliarde Menschen (könnten) in die Armut rutschen“ (Frankfurter Rundschau 2020c, S. 14). Doch zugleich, so das Entwicklungshilfswerk, „verdienten 32 der profitabelsten Unternehmen der Welt im Gesamtjahr 2020 trotz der Corona-Krise Schätzungen zufolge insgesamt 109 Milliarden US-Dollar mehr als 2019.“ (Oxfam 2020, S. 3 f.) Daher erstaunt es gar nicht mehr, dass der weltweite „Club der Millionäre“ wächst, die Reichen weltweit hinzugewinnen (Frankfurter Rundschau v. 09.07.2020; siehe Frankfurter Rundschau 2020c) und auch in Deutschland das oberste Prozent alleine ein Drittel und das oberste Zehntel insgesamt zwei Drittel aller Vermögen sein Eigentum nennen darf, ohne dafür nennenswerte Erbschafts- oder gar Vermögensteuern abführen zu müssen (vgl. Schröder u. a. 2020, in: DIW-Wochenbericht 29/2020, S. 511 f.). Wenn schon die Armut wächst, scheint es fast banal zu wissen, dass der Reichtum auch ansteigt. Allen, die über mangelnde öffentliche Ausgaben und leere Kassen sowie sinkende Einkommen jammern, sei deshalb gesagt: An fehlendem Reichtum in unseren extrem reichen Gesellschaften liegt es auf jeden Fall nicht, dass wir mit steigender (öffentlicher) Armut zu rechnen haben.

Während in Deutschland im September 2020 immer noch über sechs Millionen Beschäftigte in Kurzarbeit sind (vgl. Frankfurter Rundschau 2020b) und damit enorme Einkommenseinbußen zu erwarten haben, waren einige Krisen-Profiteure bereits zu Beginn des Corona-Crashs in bester Laune. Denn nicht alle leiden unter den Corona-bedingten Wirtschaftsabstürzen. Finanzspekulanten wie der Fonds-Manager Hendrik Leber können dem Börsencrash viel abgewinnen. Auf die Frage von Focus Online, ob man sich als sog. Value-Investor über einen Börsencrash freue, antwortete der sog. Finanzprofi: „Ja, ich habe meinem Team gesagt, lasst uns auf die Jagd gehen. Denn uns kommen reihenweise tolle Gelegenheiten entgegen, Aktien, die bisher immer zu teuer waren. Ich bin in einer richtig positiven Stimmung!“ (Focus 2020).

Der Paritätische Gesamtverband empörte sich derweil über das angebliche Versagen des Marktes und bemerkte nicht, dass er mit seiner Kritik das Grundprinzip kapitalistischer Märkte offenbart hat. „Wenn eine Atemschutzmaske Mitte Februar noch nicht einmal 50 Cent und sechs Wochen später 13 € koste, sei dies ein Lehrbuchbeispiel für Marktversagen“, so der Paritätische Gesamtverband in einer Pressemitteilung vom 30. März 2020  (siehe Paritätischer Gesamtverband 2020). Es ist aber eher ein Lehrbuchbeispiel für die Gesetze des Kapitalismus. Das ist kein Versagen des Marktes – so funktioniert er. So funktioniert Kapitalismus, der gerade in Krisenzeiten sein absolut zynisches Wesen offenbart. Wem das nicht gefällt, der und die muss sich für eine gemeinwohlorientierte Wirtschaftsweise mit vorrangigem Gemeineigentum einsetzen, welche profitorientierten Kapitalismus hinter sich lässt. Wie es Gabriele Winker formuliert, ist „der Zweck einer kapitalistischen Ökonomie (…) die Verwertung des eingesetzten Kapitals. Dafür muss Arbeitskraft in hinreichender Quantität und Qualität zur Verfügung stehen. Dies wird in einer kapitalistischen Gesellschaft primär unentlohnt durch die Sorgearbeit in Familien gewährleistet. Hier wird die zukünftige Generation der Erwerbstätigen geboren, erzogen und betreut, und hier wird auch die Arbeitskraft der derzeitigen Erwerbstätigen wiederhergestellt. Seit im neoliberalen Kapitalismus alle erwerbsfähigen Personen durch Erwerbsarbeit eigenständig für ihren Lebensunterhalt aufkommen müssen, fehlt jedoch für diese familiäre Sorgearbeit die Zeit. Dieses Problem wird noch verstärkt durch eine staatliche Austeritätspolitik, die an der sozialen Infrastruktur spart, mit der Konsequenz, dass es in der öffentlichen Daseinsvorsorge fast überall an Personal und Ressourcen fehlt. Besonders sichtbar wird dies in Zeiten von Corona in den Krankenhäusern und Pflegeheimen am Mangel an Pflegekräften oder Schutzausrüstung“ (Winker 2020). Und es ließe sich noch hinzufügen, dass die (Bewältigung der) Corona-Krise die Privatisierung der Risiken und die Re-Traditionalisierung der Familien- und Geschlechterverhältnisse noch verstärkt (hat) – und das nicht nur in Deutschland (vgl. Charrel 2020; Neuhaus 2020).

Schnell wurde während der einsetzenden Wirtschaftskrise klar, dass die Folgen des Crashs vor allem von den Lohnabhängigen zu tragen sein werden. Gerade die digitalen Gewinner der Krise von Apple über Microsoft und Google bis Amazon haben sich bislang durch sog. kreative Steuergestaltung der öffentlichen Finanzierung auskömmlicher Sozialsysteme und ausreichender Gesundheitsversorgung entzogen. Und umgekehrt wurden oft die Stärksten von den Wirtschaftshilfen der Staaten am meisten bedient, sodass selbst eingefleischte Unternehmer ins Grübeln kamen: „Bei den gegenwärtigen Diskussionen um Rettungsmaßnahmen und die Übernahme von Lasten der Krise könnte ich glatt zum Sozialisten werden“, klagte der Chef der Hotelgruppe „Motel One“, Dieter Müller, in der Frankfurter Allgemeinen Zeitung v. 29.04.2020 über die „Rettungsmaßnahmen“ der Bundesregierung etwa für Banken (vgl. Köhn 2020). Große Konzerne, wie VW, Daimler, BMW, TUI usw. schütteten weiterhin Milliarden Dividenden an ihre Kapitaleigner aus, während sie hunderttausende ihrer Beschäftigten in Kurzarbeit schickten, sodass diese nicht nur gravierende Gehaltseinbußen hatten, sondern die Steuer- bzw. Beitragszahlenden einen Teil der Gehälter und der Dividenden finanzieren durften. Selbst in den schlimmsten Krisenphasen und zu den schlimmsten Krisenzeiten im Frühjahr 2020 wurde die Rüstungsproduktion mit allen Begleiterscheinungen aufrechterhalten (was zumindest einige Priester in Italien zum Anlass nahmen, dies zu kritisieren; vgl. Vaticannews.va 2020). All dies kann als Nachweis für die These gewertet werden, dass die Corona-Krise und ihre Bewältigung nicht für alle gleich wirkten, ja, dass sie die Umverteilung von unten nach oben sogar noch verstärkt haben.

## Ursachen und strukturelle Grundlagen

Wie der Kölner Politikwissenschaftler Christoph Butterwegge richtig schreibt, hat sich die wirtschaftliche, soziale und politische Ungleichheit (in Deutschland) dergestalt entwickelt, dass der viel bemühte „soziale Zusammenhalt“ schwindet und daher mit guten Gründen buchstäblich von einer „zerrissenen Republik“ gesprochen werden kann. „Armut und Reichtum sind im Kapitalismus der Gegenwart strukturell so miteinander verzahnt, dass beide tendenziell zunehmen. Die zum Teil skandalös niedrigen (Dumping‑)Löhne für Millionen prekär Beschäftigte bedeuten nämlich hohe Gewinne, Dividenden und Renditen für Unternehmer, Kapitalanleger und Börsianer“ (Butterwegge 2020, S. 110). Während also aus der sozialen Spaltung heraus im globalen Maßstab ökonomische Krisen, Kriege und Bürgerkriege resultieren, die wiederum größere Migrationsbewegungen nach sich ziehen, sind in Deutschland der soziale Zusammenhalt und die repräsentative Demokratie bedroht.

Darauf allerdings, dass die Umverteilung von oben nach unten eine Schlüsselfrage der Entwicklung moderner Gesellschaften darstellt, hatte schon der 2012 verstorbene britische Sozial- und Universalhistoriker Eric Hobsbawm am Ende des 20. Jahrhunderts hingewiesen. Auch unter Berücksichtigung ökologischer Zusammenhänge prognostizierte er 1995, dass die Politik des neuen Jahrtausends vielmehr durch soziale Umverteilung als durch ökonomisches Wachstum bestimmt sein werde. Dabei war er damals schon nicht so naiv wie manche öko-kapitalistische Forderung nach der Macht der Märkte für mehr Nachhaltigkeit, wie sie etwa in einem Interview mit den Vorsitzenden der Grünen Habeck und Baerbock formuliert werden (vgl. FAZ.net 2019). Stattdessen vermutete Hobsbawm (1995, S. 711): „Die marktunabhängige Zuteilung von Ressourcen, oder zumindest eine scharfe Beschränkung der marktwirtschaftlichen Verteilung, wird unumgänglich sein, um der drohenden ökologischen Krise die Spitze zu nehmen“. Man könne mit Sicherheit davon ausgehen, dass es mit wachsendem Wohlstand auf der Erde auch auf politischer und rechtlicher Ebene eine wachsende Ungleichheit geben werde (Hobsbawm 2000, S. 163 f.). Damit ist aber auch ein Aspekt von Macht- und Herrschaftsinteressen im Spannungsfeld von Armut und Reichtum angesprochen, welcher bei der Behandlung der Themen oft ausgeblendet bleibt.

Die Aufteilung des Reichtums wird Hobsbawm zufolge „auf dramatische Weise ungleicher“ (ebd., S. 113). Mit „dramatisch“ meint er, „daß eine sehr kleine Zahl von Menschen, häufig einzelne Individuen, in einer Weise reich werden, die mindestens seit der Zeit einer Feudalgesellschaft beispiellos ist (…)“ (ebd.). Hobsbawm hält das Ausmaß des Reichtums, der heutzutage einzelnen Personen zur Verfügung steht, für absolut unglaublich: „Global gesehen ist der Reichtum in der Hand von einem Prozent der Weltbevölkerung ungeheuer. In welcher Weise wird sich dies auf die Politik auswirken? Das ist nicht klar. Wir haben Hinweise aus den Vereinigten Staaten, wo Privatpersonen heute in der Lage sind, Wahlkampagnen für Präsidentschaftskandidaten zu führen oder mit ihren privaten finanziellen Mitteln wirksam zu beeinflussen. Heute sind die Reichen zu Dingen in der Lage, die früher nur durch große kollektive Organisationen bewerkstelligt werden konnten. Ich bin nicht sicher, ob wir die tiefreichenden Implikationen dieses Phänomens wirklich verstanden haben“ (ebd., S. 114). In den Wohlstandsländern sei noch immer die Tatsache augenfällig, „daß diejenigen, die mit einem materiellen Vorsprung geboren werden, im Lauf ihres Lebens beobachten können, daß dieser Vorsprung sich exponentiell um ein Mehrfaches vergrößert. In zahlreichen Untersuchungen hat man nachgewiesen, daß die Ärmeren früher sterben als die Reichen und im Durchschnitt weniger gesund sind als diese. Zweifellos haben auch die Reichen ihre Probleme, doch ihr relativer Vorteil im Hinblick auf die Lebenserwartung steht außer Zweifel“ (ebd., S. 208 f.).

In diese Richtung hat sich der globale Kapitalismus weiterentwickelt und es scheint nichts und niemanden zu geben, der oder die ihn aufzuhalten in der Lage sind. Viele Theorien zeitgenössischer Soziolog_innen abstrahieren indessen laut Butterwegge von sozioökonomischen Produktions‑, Eigentums- und Klassenverhältnissen als zentralem Strukturmoment bürgerlich-kapitalistischer Gesellschaften. Stattdessen vereng(t)en sie sich immer wieder stark auf die Konsumtionssphäre und deren sensationelle Angebotsfülle (Butterwegge 2020, S. 117 f.). Auch die veröffentlichte Meinung habe sich hierzulande zu keinem Zeitpunkt ernsthaft mit dem Problem der sozioökonomischen Ungleichheit auseinandergesetzt und praktisch nie reale Möglichkeiten zu seiner Lösung eruiert. „In den maßgeblichen, das Alltagsbewusstsein prägenden Medien wird der Reichtum (…) eher verschleiert und die Armut verharmlost. Dasselbe gilt für die etablierten Parteien und Politiker, deren ganzes Bestreben darauf gerichtet ist, die bestehenden Verteilungsverhältnisse und ihre eigene Mitverantwortung dafür zu rechtfertigen“ (ebd., S. 143). Zustimmend referiert Butterwegge die Position des Soziologen Jürgen Ritsert, wonach die meisten soziologischen Ansätze während der verschiedensten Phasen der BRD-Geschichte das Ziel verfolgten, die Klassen durch Theorie zum Verschwinden zu bringen. Ob die aktuellsten Diskurse über soziale Ungleichheit national und international (z. B. Thomas Piketty und Papst Franziskus) auf den Beginn einer neuen Phase der Wahrnehmung sozialer Disparitäten hindeuten, lässt er offen (vgl. Butterwegge 2020, S. 205).

## Ungleiche Corona-Folgen

Auch der Vorsitzende der Kinderkommission des Deutschen Bundestages, Matthias Seestern-Pauly (FDP), musste in einem Interview mit der ZEIT vom 16. Juli 2020 die Nicht-Beachtung der UN-Kinderrechtskonvention während der Corona-Krise klar und deutlich rügen: „Die Belange der Kinder in der Corona-Krise sind nicht ausreichend wahrgenommen worden – darüber sind wir uns in der Kommission einig. Darum haben wir in einer Erklärung angemahnt, dass die in der UN-Kinderrechtskonvention verbrieften Rechte und die Bedürfnisse der Kinder auch in der Corona-Krise nicht aus dem Blick geraten dürfen“ (Schoener 2020, S. 28).

Es war nicht zu übersehen, dass die Entscheidungen der Bundesregierung während der Pandemie das Kindeswohl nicht vorrangig berücksichtigt haben. Zentrale Schutzrechte blieben unbeachtet. Besonders davon betroffen waren und sind Kinder in Armut und in prekären Lebensbedingungen (so z. B. ca. drei Millionen Kinder im Rechtskreis des Bildungs- und Teilhabepakets, denen von heute auf morgen das kostenlose Mittagessen in der Kita- oder Schul-Einrichtung ohne Kompensation gestrichen wurde). Elementare Schutz‑, Fürsorge- und Beteiligungsrechte von Kindern und Jugendlichen sind in der Corona-Krise in Deutschland verletzt worden (so wurden Kinder, Jugendliche, Schüler_innen-Vertretungen, Jugendverbände und Kinderrechtsorganisationen überwiegend nicht gefragt oder auch nur in Kenntnis gesetzt darüber, was man mit ihnen und ihren schulischen sowie außerschulischen Betreuungs‑, Bildungs- und Betätigungseinrichtungen vorhatte). Außerdem haben die Maßnahmen zur Verstärkung von Kinderarmut beigetragen (vgl. Houben 2020). Dagegen sollten an Kinderperspektiven anknüpfende Alternativen und Gegenstrategien Konzepte der Armutsbekämpfung, der Partizipation junger Menschen und der Förderung sozialer Infrastruktur vereinen, die den gesellschaftspolitischen Kontext nicht aus den Augen verlieren (vgl. Klundt 2020, S. 15 f.).

Indessen sind die psychosozialen Folgen der Corona-Krise (und der ergriffenen Maßnahmen) laut „Copsy“-Studie (Corona und Psyche) der Hamburger Universitäts-Klinik Eppendorf (UKE) nicht übersehbar. Vor der Pandemie fühlte sich ein Drittel aller befragten Kinder und Jugendlichen „psychisch stark belastet“, seit der Corona-Krise sind es 71 %. „27 Prozent der Kinder und Jugendlichen sowie 37 Prozent der befragten Eltern gaben an, dass es mehr Streit in der Familie gab“ (Frankfurter Rundschau v. 11./12.07.2020). Diese Ergebnisse hat selbst die Forscherinnen und Forscher erstaunt, wie die Kinder- und Jugendpsychiaterin Ulrike Ravens-Sieberer vom UKE die enorme Verschlechterung des psychischen Wohlbefindens kommentiert: „Dass sie allerdings so deutlich ausfällt, hat auch uns überrascht.“ (Frankfurter Rundschau v. 11./12.07.2020, S. 3).

Und, wie auch das neuerliche „Factsheet“ der Bertelsmann-Stiftung zu „Kinderarmut in Deutschland“ von 2020 ein weiteres Mal nachgewiesen hat, sind die Entwicklungen im Bereich Kinderarmut gerade mit der Corona-Krise noch einmal verschärft worden. So wachse mehr als jedes fünfte Kind in Deutschland in Armut auf, was immerhin 2,8 Mio. Kinder und Jugendliche unter 18 Jahren betrifft. Die Kinder- und Jugendarmut verharre seit Jahren auf diesem hohen Niveau. Trotz langer guter wirtschaftlicher Entwicklung seien die Zahlen kaum zurückgegangen. Kinderarmut sei seit Jahren ein ungelöstes strukturelles Problem in Deutschland. „Die Corona-Krise wird die Situation für arme Kinder und ihre Familien weiter verschärfen. Es ist mit einem deutlichen Anstieg der Armutszahlen zu rechnen. Aufwachsen in Armut begrenzt, beschämt und bestimmt das Leben von Kindern und Jugendlichen – heute und mit Blick auf ihre Zukunft. Das hat auch für die Gesellschaft erhebliche negative Folgen“ (Bertelsmann-Stiftung 2020, S. 1). Die Vermeidung von Kinderarmut müsse gerade jetzt politisch Priorität haben. Sie erfordere neue sozial- und familienpolitische Konzepte, wozu Strukturen für eine konsequente Beteiligung von Kindern und Jugendlichen und eine Absicherung ihrer finanziellen Bedarfe gehöre (durch ein sog. Teilhabegeld oder eine Grundsicherung; vgl. ebd.).

Derweil werden vor allem Menschen mit niedrigen Einkommen in Deutschland von der Corona-Pandemie belastet. So ist laut einer Studie der Hans-Böckler-Stiftung die Zahl der Erwerbstätigen, die zwischen April und Juli 2020 Einkommenseinbußen hinnehmen mussten, von 20 auf 26 % gestiegen. Klagten jedoch bei den Haushalten mit mehr als 3200 € Nettoeinkommen 22 % über Lohneinbußen, so waren es bei den Haushalten mit einem monatlichen Nettoeinkommen unter 1500 € immerhin 40 % und bei denen mit bis zu 2600 € mit 30 % immer noch fast ein Drittel der Betroffenen (vgl. Müller 2020, S. 5).

Zugleich betont die Forscherin Shan Huang beim Deutschen Institut für Wirtschaftsforschung (DIW Berlin), dass Corona sozial Benachteiligte stärker als andere bedroht. Diejenigen werden am schwersten vom Virus betroffen, die ohnehin sozial benachteiligt seien. Wer dazu noch ethnischen Minderheiten entstamme, unterliege besonders hohen Krankheits- und dazu Mortalitätsrisiken. „In den USA ist den Centers for Disease Control and Prevention zufolge das Risiko eines Krankenhausaufenthaltes unabhängig vom Alter für sie vier- bis fünffach höher. Ähnliche Erhebungen von Public Health England ergeben, dass unter Berücksichtigung demografischer Effekte die Sterblichkeit in Großbritannien unter ethnischen Minderheiten bis zu doppelt so hoch ist“ (Huang 2020, S. 12). Auch seien laut verschiedenen US-Studien unter afroamerikanischer oder eingewanderter Bevölkerung in den USA die Testwahrscheinlichkeit niedriger, die Test-Positivraten deutlich höher und Infektionen unter sozial Benachteiligten systematisch untererfasst. Für Deutschland weise eine erste Analyse der AOK Rheinland/Hamburg und des Universitätsklinikums Düsseldorf auch in die ähnliche Richtung höherer Corona-Gesundheitsrisiken bei niedrigerem Sozialstatus – am Beispiel Erwerbslosigkeit. Demnach sei „ein erhöhtes Risiko von schweren Corona-Verläufen bei Arbeitslosigkeit“ zu erkennen (ebd.).

Auf der globalen Ebene befürchten verschiedene Hilfswerke, dass die Zahl der Hungernden auf dieser Erde wieder über eine Milliarde Menschen steigen könnte, wenn der „Brandbeschleuniger“ Corona auf die sowieso schon von Klimawandel, Konflikten und Naturphänomenen wie z. B. Heuschrecken geplagten Krisenländer des globalen Südens treffe. „Die Welthungerhilfe-Chefin warnte davor, Lockdowns als Allheilmittel in der Pandemie zu betrachten und darüber die entstehenden Kollateralschäden zu unterschätzen. So gingen die Investitionen in Entwicklungsländern zurück“ (Frankfurter Rundschau 2020a, S. 13).

Auch der Aufsatz „Internationale Soziale Arbeit neu denken“ von Ronald Lutz und Tanja Kleibl setzt sich mit der „Verschärfung Globaler Ungleichheit durch Covid-19“ auseinander (Lutz und Kleibl 2020, S. 247 ff.). Über deren sozioökonomischen und ausbeuterischen Entstehungsbedingungen machen sich die Autor_innen keine Illusionen: „Globale Ungleichheit muss als historisch gewachsenes Phänomen von Kolonialismus, Imperialismus und Kapitalismus verstanden werden“ (ebd., S. 247). Sie referieren die Erkenntnisse der Hilfsorganisationen „Brot für die Welt“ oder „Misereor“, wonach im globalen Süden mangels sozialstaatlicher Absicherungen und Infrastrukturen nach dem Lockdown „möglicherweise mehr Menschen an den Folgen der Ausgangssperre sterben werden als durch das Virus selbst“ (ebd., S. 248). Nach einigen instruktiven Beispielen aus Indien, Südafrika, Malawi, Bangladesch, Kolumbien, Brasilien zu den verwundbarsten Gruppen der jeweiligen Gesellschaften fordern Lutz und Kleibl, dass „Soziale Arbeit (…) auf allen Ebenen politischer werden“ müsse (ebd., S. 250). Ihrem diskutablen und hoffnungsfrohen Appell des Weltsozialforums „Eine andere Welt ist möglich“ ist am Ende des Aufsatzes die Forderung „eines radikale(n) Wechsel(s) in Perspektive, Sprache und Kategorien“ beigelegt, was sehr unterstützenswert scheint.

## Fazit

Somit lassen sich die Entstehungsursachen der sozioökonomischen Ungleichheit aus der häufig „Globalisierung“ genannten neoliberalen Modernisierung erklären sowie aus Fehlentscheidungen und falschen Weichenstellungen der politisch Verantwortlichen. Da die sozioökonomische Ungleichheit in den kapitalistischen Produktions‑, Eigentums- und Herrschaftsverhältnissen wurzelt und der Anstieg der Ungleichheit nicht die naturwüchsige Folge von digitaler Revolution, Wissensökonomie und kühner schöpferischer Zerstörung ist, müssen auch Konsequenzen politischer Entscheidungen mitberücksichtigt werden. Die Folgen der sozialen Ungleichheit lassen sich, wie Butterwegge hervorhebt, in der zunehmenden Polarisierung der Gesellschaft, der Prekarisierung der Lohnarbeit und der Pauperisierung eines wachsenden Teils der Bevölkerung beobachten, während auch die politische Spaltung als nicht minder problematisches Resultat der sozioökonomischen Spaltung kontextualisiert werden muss (vgl. Butterwegge 2020, S. 254 ff.).

Viele Armutsforscher_innen erforschen richtige und bedarfsgerechte Lösungen für sozialpolitische Probleme und gegen gesellschaftliche Polarisierung. Hinderungsgründe, warum diese klugen und angemessenen Vorschläge regelmäßig nicht angenommen oder angewandt werden, bleiben oft unterbelichtet. Sie erfordern die theoretisch-analytische Auseinandersetzung mit Prozessen der (Primär‑)Verteilung, der Steuerpolitik, wirtschaftlicher Interessensgegensätze und sozioökonomischer Kräfteverhältnisse insgesamt. So werden z. B. oft Anlässe für Kinderarmut, wie Scheidung, Alleinerziehenden-Status, Migrationshintergrund oder Arbeitslosigkeit mit den zugrundeliegenden Ursachen im vorhandenen Wirtschafts- und Sozialsystem verwechselt (vgl. Klundt 2019, S. 128 ff.). Dadurch gerät bei der Armutsforschung, der Sozial(arbeits)wissenschaft und bisweilen auch der praktischen Sozialen Arbeit in den Hintergrund, dass sozial gerechte Familien- und Sozialpolitik und gute Bildungs‑, Betreuungs- und Arbeitsmarktpolitik auch für Kinder von arbeitslosen, alleinerziehenden oder migrantischen Eltern ein armutsfreies Leben ermöglichen können. Der Verfasser verheimlicht nicht, dass sich eine ihm dabei notwendig erscheinende Perspektive theoretisch-praktisch mit Interessen, mit Macht und mit Herrschaft auseinandersetzen muss, also auch mit den Profiteuren der vorhandenen Ordnung, wobei Anti-Kapitalismus als buchstäblich alternativlos für einen auch nur halbwegs demokratischen und sozialen Rechtsstaat, wie ihn das Grundgesetz vorschreibt, anzusehen ist.
